# Protocol using AGO-APP for isolation and sequencing of argonaute-bound miRNAs in specific neuronal subtypes of the mouse brain

**DOI:** 10.1016/j.xpro.2026.104720

**Published:** 2026-08-04

**Authors:** Surbhi Kapoor, Andrea Erni, Harold Cremer, Christophe Beclin

**Affiliations:** 1 Aix-Marseille Université, Centre National pour la Recherche Scientifique (CNRS), Institut de Biologie du Développement de Marseille (IBDM), Marseille, France

**Keywords:** Genomics, Sequencing, RNAseq, Model Organisms, Molecular Biology, Gene Expression, Neuroscience

## Abstract

Argonaute protein affinity purification by peptides (Ago-APP) is an immunoprecipitation-based approach that isolates argonaute-bound, putatively active microRNAs (miRNAs) via expression of a T6B peptide. Here, we present a protocol for the application of Ago-APP to olfactory bulb interneurons in mice expressing a Cre-dependent T6B transgene. We outline procedures for maintaining these transgenic mice, performing T6B immunoprecipitation from brain tissue, and preparing miRNA sequencing libraries. This workflow can be applied to specific neuronal subtypes and even subcellular compartments as well as extended to other models, including *Drosophila.*

For complete details on the use and execution of this protocol, please refer to Kapoor et al.^1^

## Highlights

• Steps for preparing samples for performing Ago-APP from mouse brain tissue
• Steps for immunoprecipitating Argonaute-bound miRNA from defined mouse brain cells
• Steps for sequencing miRNA from Ago-APP samples with a low amount of material
• Highlight the advantages of Ago-APP over alternative techniques

## Abstract (editor highlights)

Publisher’s note: Undertaking any experimental protocol requires adherence to local institutional guidelines for laboratory safety and ethics.

## Graphical abstract

**Figure:**
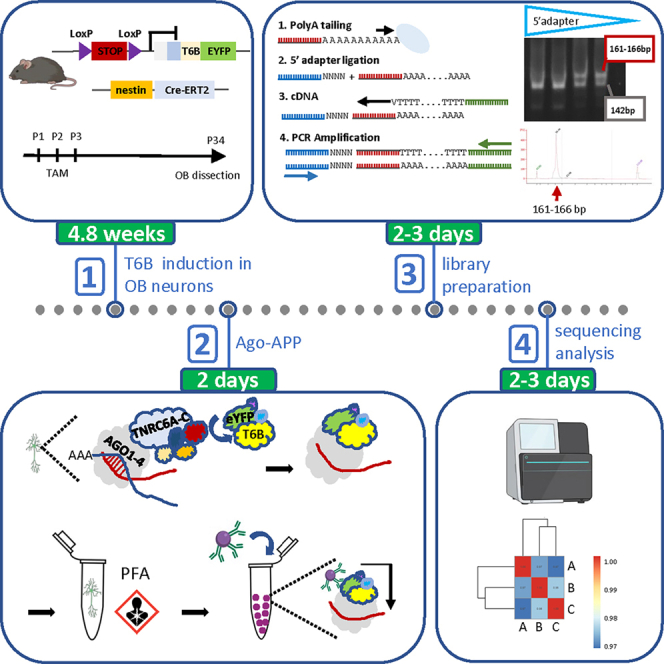

## Before you begin

Investigating active microRNAs (miRNAs) from defined neuronal populations *in vivo* remains technically challenging for several reasons. First, Neurons exhibit long branching processes and are highly interconnected, making them largely unsuitable for mechanical dissociation and cell sorting. Second not all expressed miRNAs are involved in regulatory processes^2^^,^^3^^,^^4^ defining a pool of AGO-bound, functionally engaged miRNAs and those that are inactive and non-RISC-associated. Third, miRNAs possess specific characteristics, such as their small size, high level of sequence redundancy, and lack of polyadenylation, which necessitate the adaptation of traditional protocols to enable their molecular characterization, such as sequence determination. Finally, miRNA-mediated gene regulation depends on the specific localization of miRNAs in subcellular compartments within cells. This is particularly true for neurons where miRNAs play a fundamental physiological role in regulating neuronal plasticity at the level of the individual synapse.^5^

Here, we present technical developments designed to overcome these limitations. In particular we have adapted an approach originally established *in vitro* called Argonaute Protein Affinity Purification by Peptides (Ago-APP)^6^ for an *in vivo* setting. Argonaute (Ago) proteins are the core effectors of miRNA-mediated gene silencing. They function by recruiting additional proteins to the RNA-induced silencing complex (RISC), which drives translational repression and mRNA decay through deadenylation and decapping. GW182 proteins play a central role in the process by physically linking argonaute and RISC proteins. T6B is derived from the human TNRC6B protein, a member of the GW182 family. The peptide retains the capacity to bind Ago proteins with high affinity. In the in vivo Ago-APP strategy, T6B, expressed as a FLAG-, HA- and eYFP-tagged fusion protein (T6B-YFH), competitively displaces endogenous TNRC6/GW182 proteins from the Ago–miRNA–mRNA complex without disrupting Ago–miRNA binding. The resulting T6B–Ago–miRNA complexes are then immunoprecipitated using anti-GFP agarose beads, enabling the selective isolation of functionally engaged, Ago-bound miRNAs.

A transgenic mouse line has been generated in which a CRE-inducible T6B-YFH transgene, under the control of the ubiquitous pCAGs promoter (pCAGGS-FLAGHAT6BeYFP), has been inserted at the ROSA locus.^1^ The in vivo Ago-APP protocol presented here describes how this transgenic mouse can be used for the biochemical isolation of active miRNAs from defined neuronal cell type. This approach can be extended to glial populations, and other mammalian tissues, or adapted to Drosophila melanogaster using UAS–GAL4/GAL80 systems as we recently did when analysing the collective involvement of miRNAs in neural cell development.^7^

### Innovation

This protocol introduces AGO-APP as an innovative approach for isolating functionally active miRNAs from specific neuronal populations in vivo. Traditional RNA extraction methods cannot distinguish between AGO-bound, functionally engaged miRNAs and those that are inactive and non-RISC-associated. Although strategies based on the overexpression of AGO2^8^ or the modification of the endogenous Ago2 locus^9^^,^^10^ also enable the immunoprecipitation of active miRNAs, they suffer from significant limitations. These limitations include bias toward AGO2 bound miRNA, and potential artefacts caused by the change in the expression level of the functional AGO2 protein.

The AGO-APP method overcomes these constraints through a peptide-based approach that captures all endogenous miRNAs bound to any of the four AGO proteins. The key principle of this method is the controlled expression of the T6B peptide for the specific biochemical enrichment of active miRNAs. We describe a transgenic mouse line that expresses a CRE-inducible tagged T6B transgene. Breeding this line with any Cre-expressing transgenic line enables the immunoprecipitation of AGO-bound miRNAs in any cell type. Furthermore, Ago-APP eliminates the need to sort cells to analyze their miRNA expression pattern. This is particularly advantageous for cells that are unsuited to mechanical dissociation, like mature neurons. In addition, due to its small size T6B can be fused to any protein tag to target its expression and analyze miRNA activity in specific subcellular compartments, such as the post-synaptic compartment.^1^ Finally, as AGO-APP yields limited quantities of RNA, a customized, low-input, bias-reduced small RNA library preparation workflow has been developed for next-generation sequencing.

### Institutional permissions

All mouse experiments were approved and performed in accordance with the guidelines of the French ethical committee and the European rules and regulations (Authorization number #25923-2020081214524914 v1).

Readers wishing to apply the Ago-APP technique in vivo in mice should be aware that they must first obtain approval from the relevant authorities.

### Preparation

#### Generation of the T6B x Nestin-CreERT2 transgenic mouse line

**Timing: Variable (hands-on) + >6 months animal housing**

1. Breed the transgenic mouse line with the pCAGGS-FLAGHAT6BeYFP cassette with the Nestin-CREert2 transgenic mouse line.^11^
2. Render both transgenes homozygous upon breeding.

#### Solutions

**Timing: Variable**

3. Tamoxifen stock solution (10 mg/ml).
  a. Weigh 4 mg of tamoxifen powder.
  b. Add 4 ml of 100% ethanol.
  c. Vortex the mixture for 10 s to dissolve the powder thoroughly.
  d. Add 36 ml of sunflower seed oil.
  e. Sonicate the mixture at 140 Hz for 30 min.
  f. Check the solution every 10 min during sonication and continue until it becomes completely transparent.

***Note:*** Aliquot the prepared 10mg/ml tamoxifen solution into sterile tubes. Store aliquots at −20°C until use. Avoid repeated freeze–thaw cycles.

4. Dilute a commercial 32% Paraformaldehyde (PFA) solution 1:8 in phosphate-buffered saline (PBS, pH 7.4) to obtain a fresh 4% PFA solution.

**CRITICAL:** Always prepare the fresh 4% PFA solution on the day of the experiment. Keep the 4% PFA solution on ice while using. Do not store diluted PFA for later use, as fixation efficiency decreases rapidly.

***Note:*** Perform all steps in a chemical fume hood while wearing appropriate personal protective equipment (lab coat, gloves, and safety glasses).

5. 1 M glycine solution.
  a. For 1L of 1M glycine solution, weigh 75.07 g of glycine (molecular weight = 75.07 g/mol).
  b. Transfer the glycine powder into a clean glass bottle or beaker.
  c. Add 800 ml of distilled or deionized water.
  d. Stir the solution at room temperature using a magnetic stirrer until the glycine has completely dissolved.
  e. Adjust the final volume to 1L with distilled or deionized water.
  f. Measure the pH. If necessary, adjust the pH to the desired pH 7.0 using HCl or NaOH.

***Note:*** Use sterile water for preparation and sterile filter tips, aliquot, and store at 4°C.

6. 1M KCl solution.
  a. For 1L of 1M KCl solution, weigh 74.55 g of KCl (molecular weight = 74.55 g/mol).
  b. Transfer the KCl powder into a clean glass bottle or beaker.
  c. Add 800 ml of distilled or deionized water.
  d. Stir the solution at room temperature using a magnetic stirrer.
  e. Adjust the final volume to 1L with distilled or deionized water.

***Note:*** Autoclave the solution after preparation to ensure sterility. Store the sterilized solution at room temperature. When properly prepared and handled under sterile conditions, the solution remains stable and suitable for use for up to six months.

7. 1M Tris pH7.5.
  a. For 1L of 1M Tris solution, weigh 121.14 g of Tris base (molecular weight = 121.14 g/mol).
  b. Transfer the Tris base powder into a clean glass bottle or beaker.
  c. Add 800 ml of distilled or deionized water.
  d. Stir the solution at room temperature using a magnetic stirrer.
  e. Adjust the pH to 7.5 by slowly adding concentrated 12N HCl gradually and continuously monitoring using a calibrated pH meter.
  f. Adjust the final volume to 1L with distilled or deionized water.

***Note:*** Autoclave the solution after preparation to ensure sterility. Store the sterilized solution at room temperature. When properly prepared and handled under sterile conditions, the solution remains stable and suitable for use for up to six months.

8. 0.5 mM EDTA solution.
  a. For 1L of 0.5M EDTA, weigh 186.1 g of EDTA disodium salt dihydrate (Na_2_EDTA·2H_2_O, molecular weight = 372.24 g/mol).
  b. Transfer the EDTA powder into a clean glass bottle or beaker.
  c. Add 800 ml of distilled or deionized water.
  d. Stir the solution at room temperature using a magnetic stirrer.
  e. Slowly add a 10N NaOH solution to raise the pH to 8.0.
    ***Note:*** EDTA will not dissolve completely at neutral pH.
  f. Continue stirring until the solution becomes clear and all EDTA is dissolved.
  g. Adjust the final volume to 1L with distilled or deionized water.
    ***Note:*** Autoclave the solution after preparation to ensure sterility. Store the sterilized solution at room temperature. When properly prepared and handled under sterile conditions, the solution remains stable and suitable for use for up to six months.
9. 1M Mgcl2 solution.
  a. For 1L of 1M Mgcl2 solution, weigh 203.30 g of MgCl_2_·6H_2_O (molecular weight = 203.30 g/mol).
  b. Transfer the MgCl2 powder into a clean glass bottle or beaker.
  c. Add 800 ml of distilled or deionized water.
  d. Stir the solution at room temperature using a magnetic stirrer.
  e. Adjust the final volume to 1L with distilled or deionized water.

***Note:*** Autoclave the solution after preparation to ensure sterility. Store the sterilized solution at room temperature. When properly prepared and handled under sterile conditions, the solution remains stable and usable for use for up to six months.

10. 4% SDS solution.
  a. Prepare a 0.1M NaHCO_3_ solution by dissolving 8.401 g of NaHCO_3_ NaHCO_3_ powder into 1L of distilled water.
  b. Dilute a commercial 20% SDS stock solution 1:5 with the 0.1M NaHCO_3_ solution to obtain the 4% SDS working solution.
11. 1M Dithiothreitol (DTT) solution.
  a. Weigh 1.5 g of DTT powder.
  b. Add 10 ml distilled water and dissolve completely.
12. 100 mM NaF solution.
  a. Weigh 0.42 g of NaF powder.
  b. Add 100 ml distilled water and dissolve completely.
13. Prepare a 40 mg/ml proteinase K stock solution in RNase-free water.

***Note:*** Store the stock of proteinase K solution at −20°C.

14. 1% BSA.
  a. Weigh 0.1 g of BSA powder.
  b. Add 8 ml 1X PBS, mix it well by using rocker (Do not vortex), once dissolved bring final volume upto 10 ml with 1X PBS.
  c. Aliquote the solution in 1 ml eppendrof tubes and store it in at 4°C for short-term use or aliquot and store at −20°C for long-term stability.

## Key resources table

**Table:** 

REAGENT or RESOURCE	SOURCE	IDENTIFIER
**Chemicals, peptides, and recombinant proteins**		

Sunflower seed oil	Sigma Aldrich	S5007 250ML
Tamoxifen	Sigma Aldrich	T5648-1G
Paraformaldehyde 32%	EUROMEDEX	EM-15714
Sodium Dodecyl Sulfate (SDS) 20%	EUROMEDEX	EU0660-B
SODIUM FLUORIDE (NaF)	Merck	PHR1408-1G
TBE BUFFER, 10X	Thermo Fisher Scientific	15581044
Tris-Hcl 1M pH7	Interchim SA	AYO661
TRIZOL REAGENT	Thermo Fisher Scientific	15596026
GlycoBlue Coprecipitant	Thermo Fisher Scientific	AM9515
Proteinase K (PCR grade)	Roche product	3115801001
*E. coli* poly(A) polymerase	New England Biolabs	M0276S
PHUSION HIGH-FIDELITY DNA POLY MERASE, 100U	Thermo Fisher Scientific	F530S
SuperScriptIII First Strand cDNA Super Mix	Thermo Fisher Scientific	18080–400
T4 RNA Ligase 1	New England Biolabs	M0204S
GoTaq® G2 Flexi DNA Polymerase	Promega	M7808
Na_2_EDTA	VWR	20302.236
Ethanol	VWR	20821.296
DTT (DITHIOTHREITOL)	ROTH	6908–1
GLYCINE	EUROMEDEX	26-128-6405-C
AEBSF, READY MADE SOLUTION 100 MM IN H20	Merck	SBR00015-1ML
PBS	EUROBIO	CSOPBS01-08
ethidium bromide	ROTH	2218.1
Potassium chloride (KCl)	ROTH	3904.2
Chloroform	Fisher Scientific	10488400
BSA	Merck	A7030
NP-40	Thermo Fisher Scientific	28324
MgCl_2_·6H_2_O	ROTH	A537

**Critical commercial assays**		

ChromoTek GFP-Trap® Agarose	Proteintech	gta-100
COSTAR(R) SPIN-X(R) CENTRIFUGE TUBE	Merck	CLS8163-100EA
miRCURY LNA SYBR Green PCR Kit	Qiagen	339346
miRCURY LNA miRNA PCR Assay	Qiagen	339306
6% polyacrylamide (PAA) gel- NOVEX 6% TBE GEL 1.0MM 10W	Thermo Fisher Scientific	EC6265BOX
Kit HS DNA BioAnalyzer	Agilent Technologies	5067–4626
MiniSeq High Output Reagent Kit (75-cycles)	Illumina	FC-420-1001
Pierce BCA assay kit	Thermo Fisher Scientific	23227
miRCURY LNA RT Kit	Qiagen	339340
miRCURY LNA miRNA PCR Assay for mmu-let-7a-5p	Qiagen	339306 YP00205727

**Deposited data**		

miRNA sequencing data from Ago-APP samples obtained with the T6B and PSD95-T6B expressing transgenic mouse lines	Kapoor et al.^1^	ArrayExpress: E-MTAB-15124

**Oligonucleotides**		

randomized 5′ RNA adapter GUUCAGAGUUCUACAGUCCGACGAUCNNNN	Kim et al.^12^	N/A
v-dT20-RA3 RT Primer GCCTTGGCACCCGAGAATTCCA TTTTTTTTTTTTTTTTTTTTV	Kapoor et al.^1^	N/A
TRUseq Small RNA Library Prep Kit (Indexes 1–48)	Illumina	RS-200-0012, RS-200-0024, RS-200-0036, RS-200-0048

**Experimental models: Organisms/strains**		

T6B-FHY expressing mouse lines	Kapoor et al.^1^	N/A
Nestin-CRE-ERT2 mouse line	Battiste et al.^11^	N/A

**Software and algorithms**		

R version 4.4.2	The R Foundation	RRID:SCR_001905
Package ggplot2 of R	https://ggplot2-book.org/	RRID:SCR_014601
R Package pheatmap	https://doi.org/10.32614/CRAN.package.pheatmap	RRID:SCR_016418
R Package GenomicAlignments	https://bioconductor.org/packages/release/bioc/html/GenomicAlignments.html	RRID:SCR_024236
R Package DESeq2	Love et al.^13^	RRID:SCR_016533

**Other**		

Miniseq	Illumina	RRID:SCR_016378
*Bioanalyzer 2100*	Agilent	RRID:SCR_019715

## Materials and equipment

**Table:** Lysis buffer

Reagent	Final concentration	Amount
KCl (1 M)	150mM	7.5ml
Tris pH 7.5 (1 M)	25mM	1.25ml
EDTA (0.5M)	2mM	0.2ml
ddH_2_O	N/A	41.05ml
**Total**	**N/A**	**50ml**

Store at RT for up to 3 months.

**Table:** Wash buffer

Reagent	Final concentration	Amount
NACL powder	1M	2.922g
Tris pH 7.5 (1 M)	50mM	2.5ml
Mgcl2 (1M)	5mM	0.25ml
ddH_2_O	N/A	47.25ml
**Total**	**N/A**	**50ml**

Store at RT for up to 3 months.

**Table:** PK buffer

Reagent	Final concentration	Amount
Tris pH 7.5 (1 M)	100mM	5ml
NaCl	50mM	0.146g
EDTA(0.5M)	10mM	1ml
ddH_2_O	N/A	43.5ml
**Total**	**N/A**	**50ml**

Store at RT for up to 3 months.

**Table:** Elution buffer for library preparation

Reagent	Final concentration	Amount
NACL powder	300mM	0.8766g
EDTA (0.5 M)	2mM	200μl
ddH_2_O	N/A	49.8ml
**Total**	**N/A**	**50mL**

Make it fresh for library preparation.

**Table:** RSB buffer for library prepartion

Reagent	Final concentration	Amount
Tris pH 8.5 (1 M)	10mM	0.5ml
Tween 20	0.1%	50 μl
ddH_2_O	N/A	49.5ml
**Total**	**N/A**	**50ml**

Make it fresh for library preparation.

## Step-by-step method details

### Prepare sample for AGO-APP

**Timing: 5 h (hands-on) + 34 days animal housing (step 1)**

**Timing: 5–10 min per animal (step 2)**

In this article, we exemplify the application of Ago-APP to postnatally born olfactory bulb (OB) interneurons. In the mouse brain, these neurons are generated throughout life from neural stem cells located in the subventricular zone (SVZ) along the lateral ventricles. These new neurons reach their full maturity in the OB four to five weeks after the start of the differentiation process. This section describes the step-by-step procedure for generating the biological material on which Ago-APP will be performed, from the induction of T6B expression in Nestin-expressing neural stem cells to the dissection followed by the fixation of olfactory bulbs when induced neurons are mature.

1. Induce T6B-YFH expression in OB postnatal neurons of T6B x Nestin-CreERT2 mice.
  a. Inject three doses of tamoxifen intraperitoneally at a dose of 2 mg per 30 g of body weight (200μL for a 30 g animal) into T6B x Nestin-CreERT2 pups at the P1, P2 and P3 stages.
    ***Note:*** Tamoxifen injection allows the Cre-ERT2 protein to enter the nucleus, which leads to the recombination of the floxed T6B locus. For each biological replicate, all 6–7 pups from a single tamoxifen-injected litter were pooled together to ensure sufficient starting material and reduce biological variability.
    **CRITICAL:** To perform an intraperitoneal injection, use a 30G or 31G needle at a shallow angle (10–15°) in the lower right part of the abdomen. As tamoxifen is viscous due to its oil-based nature, inject slowly and keep the needle in place for five seconds before withdrawal to ensure tamoxifen retention.
  b. Wait 31 days after the final tamoxifen injection.
    ***Note:*** This time is necessary to allow the Nestin-positive cells of the SVZ, which have been induced to express T6B, to complete their differentiation into OB interneurons.
2. Dissect the Olfactory bulb.
  a. Sacrifice mice at P34 by cervical dislocation.
  b. Cut off the head, remove the skull and carefully extract the whole brain.
  c. Dissect the left and right olfactory bulbs and immediately place them in an ice-cold dish containing 1× PBS.
  d. Using a scalpel, chop the tissue into as many small pieces as possible (approximately 20–25 pieces per olfactory bulb) in ice-cold PBS.
  e. Transfer the tissue fragments to a 1.5 ml Eppendorf tube and allow the tissue to settle at the bottom of the tube.
  f. Carefully remove the PBS.

**CRITICAL:** To preserve RNA integrity, entire dissection process must be performed as rapid as possible.

3. Fixation of the sample.
  a. Add 1 ml of freshly prepared, cold 4% PFA solution to the Eppendorf tube containing the dissected olfactory bulb tissue.
  b. Fix the tissue for 10 min at 25°C with gentle shaking (1,000 rpm).
  c. Add 330 μl of 1M glycine stock solution to quench the PFA.
  d. Incubate for 5 min at 25°C upon continuous shaking at 1,000 rpm.
  e. Wash samples twice with ice-cold PBS for 5 min each at 25°C, 1,000 rpm.

**Figure 1:**
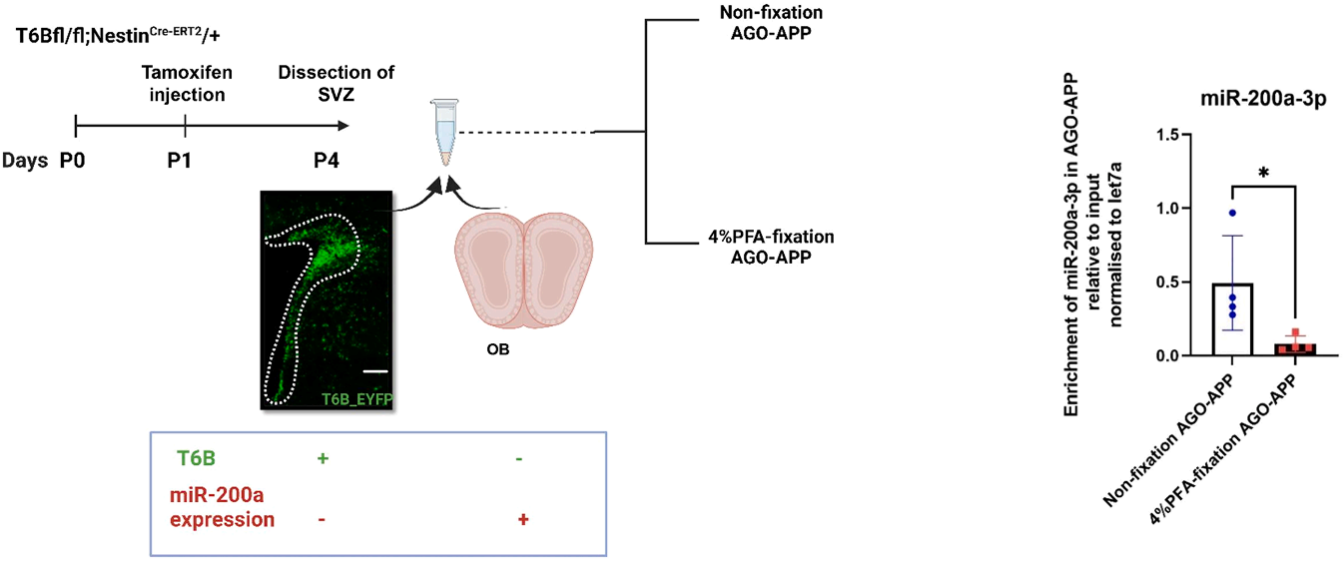
PFA fixation is required for guaranteeing the specificity of Ago-APP Three days after tamoxifen injection in T6B/nestin-CREert2 mice, T6B expression is confined to the progenitors of the SVZ. In contrast, miR-200a is absent from the SVZ but highly expressed in the OB.^12^ QRT-PCR analysis of Ago-APP samples performed on a mixture of both tissues only shows amplification of miR-200a when the SVZ tissue has not been fixed with paraformaldehyde. The histogram bars represent the mean of 4 independent replicates, Error bars: ± SEM. Mann-Whitney test: ∗ *p*-value < 0.05. Scale bar: 150 μm.

**Pause point:** Remove PBS, seal the tube, and place it in a dry ice/ethanol bath for 1 min to rapidly freeze the tissue. Store the frozen tissue at −80°C for up to six months or longer.

**CRITICAL:** The tissue is fixed with paraformaldehyde. This step is important because it stabilizes the T6B-AGO-miRNA association. When this step is omitted, miRNAs from cells that do not express T6B can displace the initial miRNAs in the T6B-AGO-miRNA complex assembled in the neural stem cells of the SVZ (Figure 1).

**CRITICAL:** Paraformaldehyde is toxic, wear gloves, mask and dispose properly.

### AGO-APP

**Timing: 2 days**

In this section, we describe the step-by-step Ago-APP procedure, which involves pulling down the miRNAs bound to AGO and T6B. To achieve this, we use beads coupled to anti-GFP antibodies, since in the transgenes expressed by the induced mice, T6B is fused to the eYFP protein.

During Ago-APP, the miRNAs that are bound to AGO and T6B are pulled down. For this purpose, because T6B is fused to the eYFP protein, we use beads coupled to anti-GFP antibodies.

4. Lysis of tissue by sonication.
  a. Prepare active lysis buffer by supplementing the standard lysis buffer with NaF, DTT and AEBSF (each at a final concentration of 1 mM) and NP-40 (final concentration of 0.01%).
  b. Remove PBS from the eppendorf tube containing the fixed tissue, add 1 ml of active lysis buffer and place the tube in an ice-water bath using a holder.
  c. Lyse the tissue at 4° by sonication.
  d. Inspect the lysate and repeat sonication until tissue pieces are no longer visible.
  e. Clarify the lysate by centrifugation at 15,000 × *g* for 15 min at 4°C.
  f. Transfer the supernatant to a fresh tube and store at −20°C.

***Note:*** For sonication, we use a Vibra-Cell sonicator with the following settings: Total time: 1 min 20s. ON/OFF pulses: 9.9/9.9s. Amplitude: 34%.

***Note:*** NaF, DTT and AEBSF are stocked at −20°C and NP-40 at room temperature.

**Pause point:** The protocol can be paused at this step by storing the lysate at −20°C for a few months.

5. Determine protein quantification in the lysate using the Pierce BCA assay kit according to the supplier (https://documents.thermofisher.com/TFS-Assets/LSG/manuals/MAN0011430_Pierce_BCA_Protein_Asy_UG.pdf).
6. Dilute the lysate to a final concentration of 2,000 μg in 1,000 μl.
7. Reserve 50 μl of lysate (100 μg of protein, 5% of total) as the input sample and keep it on ice.
8. Immunoprecipitation (IP).
  a. For each IP, use 25 μl of GFP-TRAP® Agarose (GTA) beads (Chromotek).
  b. Add the beads to an Eppendorf tube containing 1 ml of lysis buffer.
  c. Centrifuge at 2,500 *g* for 2 min at 4°C to settle the beads.
  d. Block the beads with 1% BSA for 2 h at 4°C on a rotator.
  e. Wash the beads twice with ice-cold PBS or lysis buffer, centrifuging for 2 min at 2,500 *g* between washes.
    ***Note:*** Blocked beads can be stored at 4°C for up to 6 months and used as a stock for IPs.
  f. Add the remaining cleared lysate (950 μl, representing 1,900 μg of protein) to the washed GFP-TRAP beads.
  g. Incubate for 1 h at 4°C with gentle rotation to allow the anti-GFP beads to bind the YFP-tagged T6B.
    ***Note:*** Keep the beads with the lysate at 4 °C or on ice; do not leave them at room temperature.
9. Wash the beads to remove unbound proteins.
  a. Pre-coat one Eppendorf tube per sample with ice-cold PBS for at least 1 h on ice.
  b. Prepare active wash buffer by supplementing lysis buffer with freshly added 1 mM NaF, 0.01% NP-40, 0.1 mM DTT, and 0.1 mM AEBSF. For a single IP (one Eppendorf tube), use 4 ml of active wash buffer to perform four washes.
  c. Centrifuge the tubes containing the beads at 2,500 *g* for 2 min at 4°C.
  d. Discard the supernatant.
  e. Add 1 ml of active wash buffer to the GFP-TRAP beads and centrifuge at 2,500 *g* for 2 min at 4°C and discard the supernatant. Discard the supernatant.
  f. Repeat this washing step three times.
  g. Perform a final wash with ice-cold 1× PBS.
  h. Transfer the beads to a pre-coated tube.
  i. Centrifuge at 2,500 *g* for 2 min at 4°C.
  j. Wash the beads once more with 1× PBS.
  k. Centrifuge at 2,500 *g* for 2 min at 4°C.
  l. Carefully remove the PBS, leaving only the beads at the bottom of the tube.

***Note:*** All the steps are performed at 4°C.

***Note:*** The pre-coating of Eppendorf tubes prevents bead adhesion.

10. Detachment of T6B–AGO–miRNA Complex from GFP Beads.
  a. Prepare 150 μl of 4% SDS for each IP.
    ***Note:*** Do not keep the SDS solution on ice otherwise it will flocculate.
  b. Add 50 μl of 4% SDS to the input sample and each IP sample.
  c. Incubate for 10 min at 50°C while shaking at 700 rpm.
  d. Maintain the input sample containing 50 μl lysate plus 50 μl 4% SDS at room temperature.
  e. Centrifuge the IP samples, at 2,500 *g* for 5 min at 4°C.
  f. Collect the supernatant containing the detached T6B–AGO–miRNA complex into a new tube.
  g. Repeat the elution by adding another 50 μl of 4% SDS to the beads.
  h. Incubate for 10 min at 50°C while shaking at 700 rpm.
  i. Centrifuge at 2,500 *g* for 5 min at 4°C.
  j. Collect this supernatant and combine it with the previous one.
11. Reverse Crosslinking of T6B–AGO–miRNA Complex and miRNA release.
  a. Dilute the Proteinase K stock solution in Proteinase K (PK) buffer to obtain a 20 mg/ml working solution.
  b. Pre-incubate the Proteinase K working solution for 20 min at 37°C to inactivate potential RNases.
  c. Add 100 μl of the Proteinase K working solution to both the input and IP samples.
  d. Incubate for 2 h at 65°C while shaking at 1,000 rpm.
12. RNA Extraction and Precipitation.
  a. Add 1 ml of TRIzol to the samples.
  b. Incubate for 3 min at room temperature.
  c. Add 200μL of chloroform.
  d. Vortex for 1-2 min.
  e. Centrifuge at 12,000 *g* for 20 min at 4°C.
  f. Transfer the aqueous phase to a new 2 ml tube.
  g. Add 1 μl of GlycoBlue and 1.25 volume of ice-cold isopropanol to the aqueous phase.
  h. Incubate overnight at −20°C.
  i. Centrifuge the samples at 12,000 *g* for 20 min at 4°C.
  j. Wash the RNA pellet with ice-cold 70% ethanol for 5 min.
  k. Centrifuge at 7,500 *g* at 4°C.
  l. Air-dry and dissolve in 12 μl RNase-free water.

***Note:*** Safety precautions when using TRIzol and/or chloroform: Only work in a fume hood. Wear double nitrile gloves and splash goggles. Use sealed centrifuge rotors. Flush any skin contact immediately with PEG, followed by water.

### Library preparation and miRNA sequencing of the AGO-APP samples

**Timing: 4–6 days**

Protocols for preparing a miRNA sequencing library are commonly based on the ligation of RNA adapters at the 3′ and 5′ ends of the miRNA (RA3 and RA5 adapters in the Illumina TRUseq kit). However, it has been reported, and we have also verified in our laboratory, that this step introduces strong bias, whereby some miRNAs are more accessible to adapter ligation than others.^12^ Furthermore, the RNA ligation reaction is inefficient when the amount of material is low.

To circumvent these problems, we have designed an alternative procedure which we describe in this section. In this protocol, the 3′ end of the miRNA is poly-adenylated instead of being ligated to an adapter. The 5′ end of the miRNA is ligated to an RNA adapter. However, to overcome the ligation bias, the 5′ adapter is constituted of the Illumina RA5 adapter to which four random nucleotides have been added at its 3′ end^12^ and the ligation reaction composition is supplemented with PEG^12^ for molecular crowding.

**Figure 2:**
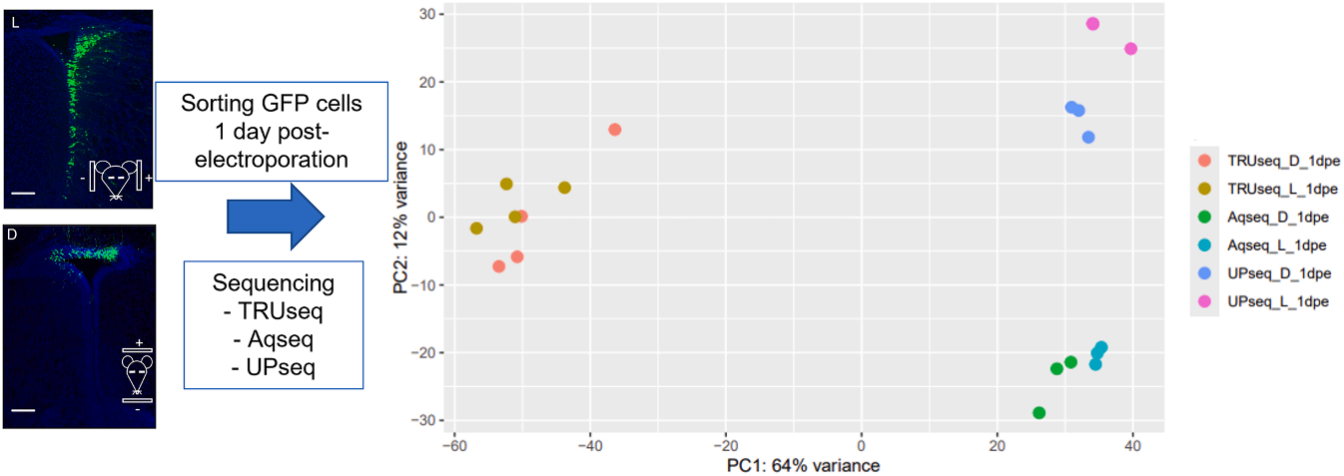
PCA analysis for comparing our sequencing method UPseq with both TRUeq and Aqseq methods We performed a Principal Component Analysis (PCA) on the sequencing results obtained using three different library preparation methods: TRUseq, Aqseq and UPseq. As shown on the left part of the figure all three methods were applied to FACS-sorted progenitor cells from the lateral (L) or dorsal (D) subventricular zone (SVZ) one day after in vivo electroporation of a green fluorescent protein (GFP) plasmid.^14^ Total RNA was extracted from the sorted cells, with RNA molecules between 15 and 40 bp selected on a gel prior to library preparation. The analysis shows that, whereas the Aqseq and UPseq data align on the PC1 axis, the TRUseq results differ significantly from those of the other two methods. Scale bars: 150 μm.

***Note:*** The reverse transcription primer is designed as follows: a V random nucleotide (which can be A, G or C), a stretch of T and the complementary sequence of the Illumina RA3 adapter. Consequently, the resulting cDNA contains the RA5 and RA3 adapter sequences at both ends.

Figure 2 shows a PCA analysis comparing several methods of sequencing library preparation: The commercial Illumina TRUseq protocol, the Aqseq protocol^12^ designed to solve the bias induced by the TRUseq method and our method that we call UPseq.

13. qPCR-based estimation of the miRNA quantity in the Ago-APP samples.
  ***Note:*** The low concentration of RNA in the Ago-APP samples precludes the use of traditional RNA quantification methods. To estimate the miRNA concentration, we quantify by qRT-PCR the let-7a miRNA which is stably and reasonably expressed in the mouse forebrain tissue. The result of this analysis will be used to set the 5′ adapter concentration in the ligation reaction (see further, step 15). For other types of tissues or cells, it may be necessary to estimate miRNA concentration using a miRNA other than let-7, provided that this miRNA is stably expressed at a sufficient level in that type of tissue or cell.
  a. Perform the reverse transcription reaction on 3 μl of the Ago-APP sample using the miRCURY LNA RT Kit in conditions described in the tables below.

**Table:** Reverse Transcription reaction master mix

Reagent	Amount
5× miRCURY RT Reaction Buffer	2 μl
10x miRCURY RT Enzyme Mix	1 μl
ddH_2_O	4 μl
miRNA	3 μl

**Table:** Reverse Transcription conditions

Steps	Temperature	Time	Cycles
Reverse transcription step	42°C	60 min	1
Inactivation of reaction	95°C	5 min	
Hold	4°C	forever
  b. Dilute the resulting cDNA 1:10 in nuclease-free water.
  c. Perform qPCR using the miRCURY LNA SYBR Green PCR Kit.

**Table:** q-PCR reaction master mix

Reagent	Amount
2x miRCURY SYBR® Green Master Mix	5 μl
ROX Reference Dye	0.5 μl
ddH_2_O	0.5 μl
miRCURY LNA miRNA PCR Assay (let-7a-5p)	1 μl
cDNA template (diluted 1:10)	3 μl

**Table:** q-PCR cycling conditions

Steps	Temperature	Time	Cycles
PCR initial heat activation	95°C	2 min	–
Denaturation	95°C	10 s	40
Annealing/Extension	56°C	1 min	
Melting curve	65-95° every 0.5°	10 s at each temperature	–
Hold	4°C	Forever
14. Polyadenylation of miRNA.
  a. Combine 9 μl of AGO-APP sample with 2 μl of 10× Poly(A) buffer, 2 μl of 10 mM ATP, 1 μl of *E. coli* polymerase, and 6 μl of nuclease-free water.
  b. Incubate the mixture at 37°C for 30 min.
  c. Deactivate the enzyme by heating at 65°C for 20 min to stop the reaction prior to miRNA precipitation.
  d. Add 80 μl of nuclease-free water and 10 μl of 5M NaCl.
  e. Add 300 μl of 100% ethanol and 1 μl of GlycoBlue™ coprecipitant (15 mg/ml).
  f. Incubate the mixture at −80°C for 1 h to precipitate the miRNA.
  g. Centrifuge at 12,000 × *g* for 30 min at 4°C.
  h. Wash the pellet with 75% ethanol.
  i. Centrifuge at 7,500 × *g* for 5 min at 4°C°C.
  j. Air-dry the pellet for 5–10 min and dissolve it in 2.5 μl of nuclease-free water.

**Pause point:** The protocol can be paused at this stage by storing the polyadenylated miRNA at 4°C for one day.

15. Ligation of the 5′ Adapter.
  a. Prepare a premix containing 1 μl of 10× T4 RNA ligase reaction buffer, 1 μl of ATP (10 mM), 4 μl of 50% PEG 8000, and 0.5 μl of randomised 5′ RNA adapter (see table below for the concentration).

**Table:** 5′ adapter concentration as a function of the concentration of miRNA in the Ago-APP sample

Ct of qRT-PCR (see step 13)	Randomised 5′ RNA adapter
10 ng/μl 0.05μM	10 ng/μl 0.05 μM
14-17 0.18μM	14-17 0.18 μM
18-19 0.05μM	18-19 0.05 μM
19-20 0.01μM	19-20 0.01 μM
21-25 0.003μM	21-25 0.003 μM
  b. Mix the premix with 2.5 μl of polyadenylated miRNA from the previous step.
  c. Heat the mixture at 90°C for 60 s.
  d. Add 1 μl of T4 RNA Ligase 1 and incubate at 37°C for 1 h.
  e. Place the reaction on ice.
    **Pause point:** The protocol can be paused at this stage by storing the polyadenylated miRNA with RA5 adapter at 4°C for one day.
    **CRITICAL:** The 5′ RNA adapter concentration must be adjusted according to the quantity of miRNAs in the Ago-APP sample. As shown in Figure 3, if the miRNA concentration is low compared to that of the 5′ RNA adapter, preferential adapter dimer amplification occurs during PCR amplification of the sequencing library. We interpret the observed adapter-dimer amplification as evidence that the four random nucleotides at the 3′ end of the 5′ adapter provide an unintended annealing platform for the reverse-transcription primer. Under these conditions, unligated 5′ adapters remaining in the reaction mixture can anneal to the primer and thus compete with genuine miRNA-containing molecules during reverse transcription.

**Figure 3:**
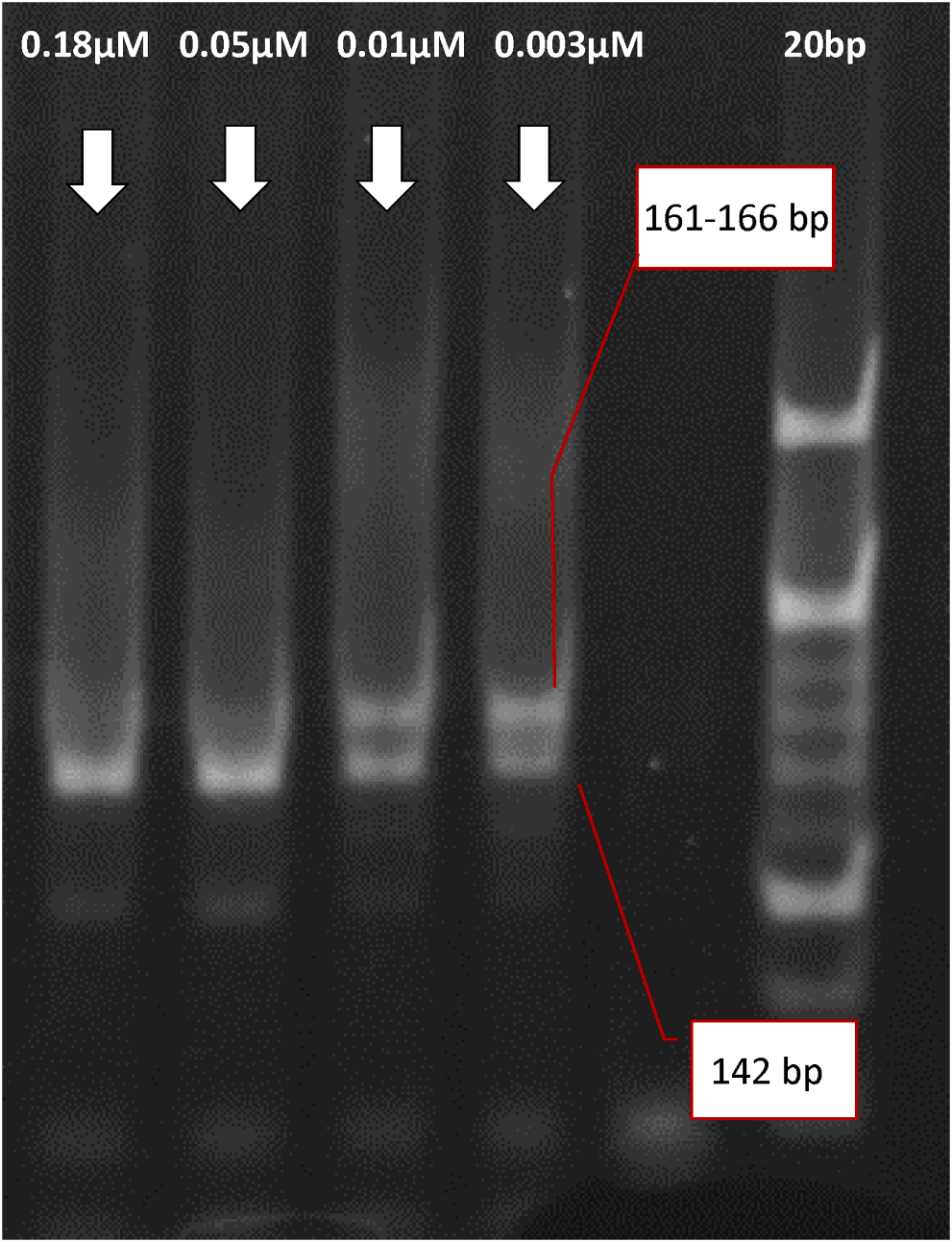
The 5′adapter concentration needs to be adapted to the amount of miRNA in the Ago-APP sample to sequence If the amount of miRNA in the Ago-APP samples is low, the use of a highly concentrated 5′ adapter can lead to the preferential amplification of the adapter-adapter dimer band.
16. cDNA Synthesis using the reaction and cycling conditions described in tables below.

**Table:** Reverse PCR reaction master mix

Reagent	Amount
polyadenylated miRNA ligated with RA5 adapter	3 μl
RT Primer (v-dT20-RA3) (100μM)	0.5 μl
Annealing Buffer	1 μl
65°C for 5 min, then place on ice.	
First Strand cDNA Super Mix	5 μl
SuperScript III enzyme	1 μl

**Table:** Reverse PCR cycling conditions

Steps	Temperature	Time	Cycles
Reverse transcription step	50°C	50 min	1
Inactivation of reaction	85°C	5 min	
Hold	4°C	forever

**Pause point:** The protocol can be paused at this step by storing the cDNA at −20°C for up to one month.

17. Perform a pilot PCR using the reaction composition mixture and cycling conditions specified in the tables below.

**Table:** Pilot PCR reaction master mix

Reagent	Amount
5x Phusion HF buffer	2 μl
5′ PCR Primer (TRUseq RP1 Primer)	0.4 μl
0.4 μl of the desired RPI? Illumina Index-Primer	0.4 μl
10 mM dNTP mix	0.25 μl
Phusion™ High-Fidelity DNA Polymerase (2U/μL))	0.1 μl
cDNA template	1 μl
nuclease-free water	5.58 μl

**Table:** Pilot PCR cycling conditions

Steps	Temperature	Time	Cycles
Initial denaturation	98°C	1 min	–
Denaturation	98°C	10 s	Check 18,21,25 cycles
Annealing	58°C	10 s	
Extension	72°C	30 s	
Final extension	72°C	10 min	–
Hold	4°C	indefinitely	–

***Note:*** The purpose of the pilot PCR is to determine the minimum number of cycles required to produce a band between 161 and 166 bp of sufficient intensity on the gel. This band corresponds to fragments containing the miRNA.

**CRITICAL:** Do not exceed 25 cycles, as this can result in an excessive number of point mutations, high duplication rates and GC bias. If 25 cycles are insufficient, address upstream issues such as poor input quality, over-fragmentation or low ligation efficiency*.*

18. Gel Electrophoresis.
  a. Mix 10 μl of O’RangeRuler 20 bp DNA Ladder with 2 μl of 5x Phusion HF buffer to adjust the salt concentration to that of the PCR samples.
  b. Add the loading buffer to the ladder and PCR samples.
  c. Load the ladder and PCR samples onto a 6% polyacrylamide (PAA) gel.
  d. Run the gel at 145V in 1× Tris-borate-EDTA (TBE) buffer.
  e. Stain the gel with ethidium bromide for approximately 5 min in 1× TBE buffer.
  f. Visualize the DNA bands under UV light and capture an image for analysis.
  g. Search for the desired miRNA containing band at 161-166 bp.

***Note:*** A band at 142bp may also be visible on the gel. This band is thought to correspond to adapter–adapter dimers. Our interpretation about the origin of these adapter-adapter dimers is discussed in the critical point of step 15.

19. Perform Scale-PCR using the reaction composition mixture and cycling conditions specified in the tables below.

Scale-PCR: Barcoding each sample with specific Index primers.

**Table:** Pilot PCR reaction master mix

Reagent	Amount
5x Phusion HF buffer	10 μl
5′ PCR Primer (TRUseq RP1 Primer)	2 μl
0.4 μl of the desired RPI Illumina Index-Primer	2 μl
10 mM dNTP mix	1.25 μl
Phusion™ High-Fidelity DNA Polymerase (2U/μL))	0.5 μl
cDNA template	5 μl
nuclease-free water	29.25 μl

**Table:** Pilot PCR cycling conditions

Steps	Temperature	Time	Cycles
Initial denaturation	98°C	1 min	–
Denaturation	98°C	10 s	Depending pilot PCR
Annealing	58°C	10 s	
Extension	72°C	30 s	
Final extension	72°C	10 min	–
Hold	4°C	indefinitely	–

***Note:*** The purpose of this scale-PCR is to amplify the sequencing library, contained within a band at 161-166bp. This band contains fragments composed of 3′ and 5′ adapters, an index sequence for multiplexing, the sequencing primers, and miRNAs of variable length (19-24nt).

20. Gel Electrophoresis and Elution of the 161-166 bp miRNA library band.
  a. Proceed with the electrophoresis as described in the “gel electrophoresis” (step 18).
  b. Visualize the DNA bands by exposing the gel to UV light and capture an image for analysis. Cut and isolate the band at 161-1660 bp from the gel.
    ***Note:*** Avoid cutting the 142 bp band which corresponds to the adapter-adapter sequence. This band does not contain any miRNA sequence but it can compete with the miRNA-containing fragments during the sequencing process (see further in step 21).
  c. Puncture the bottom of 0.5 ml Eppendorf tubes 2-3 times with a gauge needle, such as Neolus 21G with a diameter of 0.8 mm.
  d. Place each punctured Eppendorf tube into a 1.5 ml collection tube.
  e. Transfer the gel pieces into the punctured 0.5 ml Eppendorf tubes and seal them.
  f. Centrifuge the collection tubes at full speed (17,000 *g*) for 2 to 3 min to shred the gel pieces completely and allow the liquid sample to pass from the 0.5 ml tubes to the 1.5 ml tubes.
  g. Discard the smaller tubes and add 300 μl of elution buffer to each collection tube.
  h. Incubate overnight at 25°C while shaking at 800–1,000 rpm.
  i. Transfer the eluate/gel mixture onto a SpinX centrifuge filter. Spin for 2 min at 14,000 rpm to separate the DNA in the pass-through from the gel retained in the filter.
  j. Transfer the eluate into a no-retention/low-binding 1.5 ml tube. Precipitate the DNA by adding 2.5 volumes of 100% ethanol and 1 μl of GlycoBlue. Incubate for at least 30 min at −20°C. Centrifuge for 30 min at full speed at 4°C.
  k. Discard the supernatant and wash the pellet with 75% ethanol.
  l. Leave to air-dry on the bench for several min.
  m. Dissolve each pellet in 20 μl of nuclease-free water. Store at −20°C not more than two weeks.
    ***Note*:** Polyacrylamide gels are very resolutive but are fragile and prone to breaking during manipulation. To avoid this problem, this type of gels can be replaced by highly concentrated agarose gels (4%). In this case the DNA contained into the 161-166bp agarose band can be eluted using standard DNA elution kits.
21. Sequencing.
  a. Analyse the DNA content of the samples issued from 8 in a size-dependent way using the Agilent Bioanalyzer 2100 apparatus, according to the supplier’s protocol (https://www.agilent.com/cs/library/usermanuals/public/2100_Bioanalyzer_Expert_USR.pdf?srsltid=AfmBOoprZCyh_rUM5OnxW2Yo4mMLrUGCav-IuBoR-TeF2d3IZavcxSmD).
    ***Note:*** The DNA issued from the scale-up PCR and purified as described in ‘8’ mostly contains miRNA-containing molecules that have to be sequenced. However, contamination by the adapter-adapter dimer molecules may occur when cutting the bands from the gel. Moreover, the different samples will be multiplexed for sequencing in a single run. For these reasons, before mixing the different samples for sequencing a precise size-dependent DNA quantification analysis is performed in order to make sure that the different samples only marginally contain adapter-adapter dimers and that the different samples are equally represented in the multiplex.
  b. On the day of sequencing thaw the cartridge in advance in water for at least 1 h.
    ***Note:*** Take care not to submerge the cartridge by keeping the water level low.
  c. Dilute each sequencing sample in RSB buffer (provided by the supplier) to reach a concentration of 10 nM.
  d. Mix all the samples together.
  e. Dilute the 10 nM pool tenfold in RSB.
    ***Note:*** The remaining 10nM samples can be stored at −20°C and used later if the sequencing run needs to be performed again.
  f. Combine 5 μl of the 1 nM library pool with 5 μl of 0.1N NaOH and 5 μl of 200 mM Tris HCL pH7.
  g. Vortex the mixture and centrifuge to collect all the liquid at the bottom of the tube.
  h. Incubate at room temperature for 5 min.
  i. Dilute the denatured library to 5 picomolar in pre-chilled hybridization buffer (provided by Illumina) and then to 1.4 picomolar by combining 140 μl of the 5 picomolar denatured library pool with 360 μl of pre-chilled hybridization buffer.
  j. Vortex the resulting 500 μl mixture and load into the MiniSeq High Output Reagent Kit (75-cycles) cartridge.
  k. Perform the sequencing.
    ***Note:*** In our lab, we use an Illumina Miniseq apparatus for sequencing miRNAs. However, this can be done with any other sequencing machine. To configure the run settings on the Miniseq, use the Local Run Manager application. This involves naming the run, selecting the module and specifying single-read sequencing, as well as setting the number of cycles to 76. You also need to list all the samples of the multiplex associated with the appropriate index primer. Once configured, save and initiate the run. The runtime exceeds 6 h.
22. Processing of raw data and analysis.
  a. Trim the adapters from the read sequences by selecting the “Adapter Trimming option” in the Run Setting Options.
    ***Note:*** This step allows the Miniseq device to automatically trim the RA5 and RA3 sequences from each read.
  b. Trim the remaining sequences added during the generation of the sequencing library (the four random nucleotides brought by the 5′ adapter and the poly A stretch added during reverse transcription), using the ‘Trim Galore’ tool on the Galaxy.org website.
  c. Map the trimmed reads on the mm10 version of the mouse genome using the “Map with BWA” tool again on the Galaxy.org website. Specify the “single fastq“ option.
  d. Determine the number of reads per miRNA from the resulting BAM files using the GenomicAlignments package in R.

## Expected outcomes

The protocol described in this article provides detailed instructions for the isolation and sequencing of all AGO-bound miRNAs in T6B-expressing cells.

**Figure 4:**
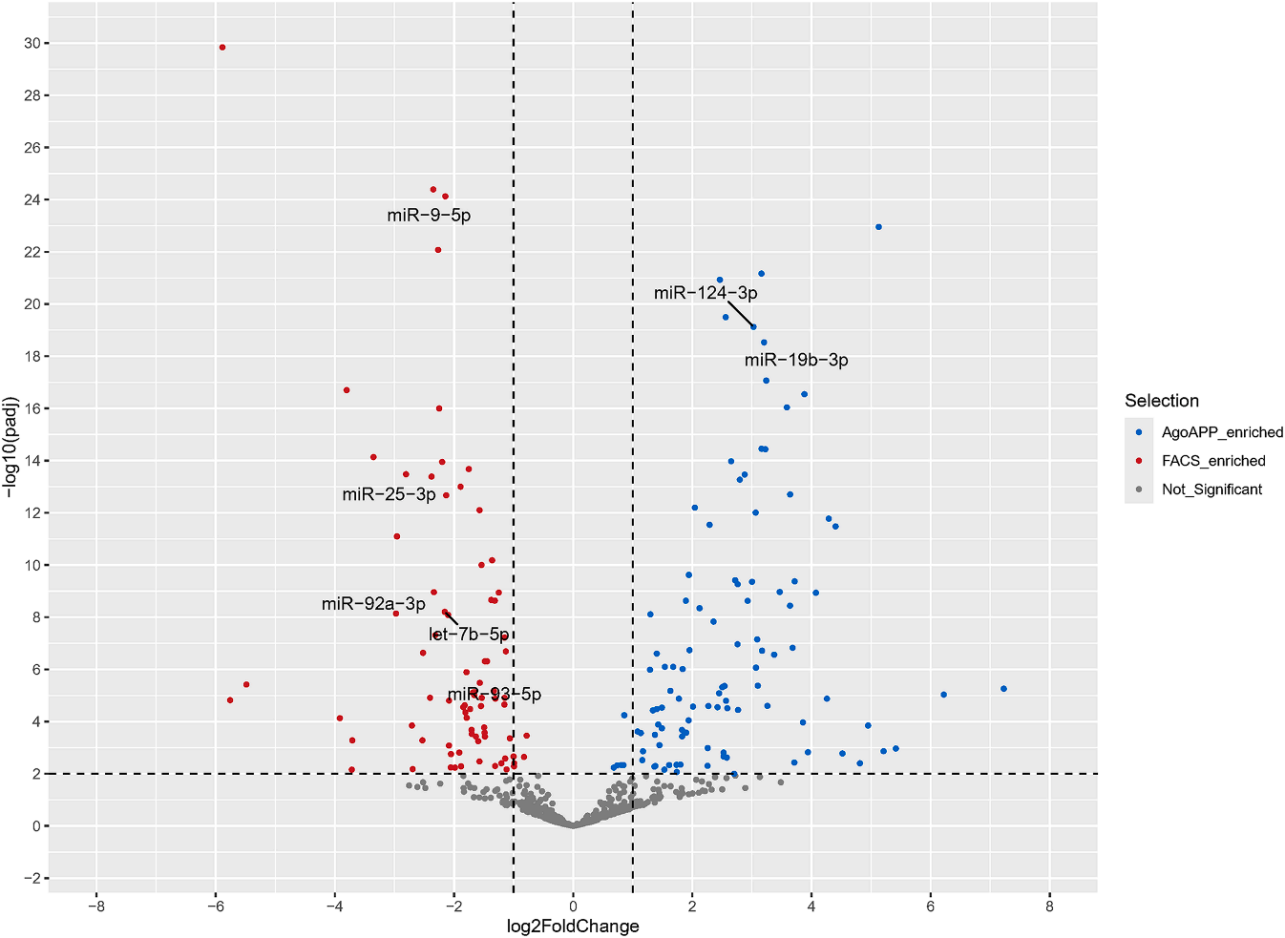
Not all expressed miRNAs are AGO-bound This volcano plot illustrates the differential (p-adjust < 0.01) enrichment (in blue) or underrepresentation (in red) of miRNA in Ago-APP samples compared to total RNA from FACS-sorted progenitors of the subventricular zone (SVZ) of T6B/nestin-CREERT2 mice. All samples were prepared four days after tamoxifen injection. Sequencing libraries were prepared according to our UPseq protocol. For the FACS-sorted samples, an additional on-gel selection step was performed to isolate RNA molecules ranging from 15 to 40 bp in length. Labeled miRNAs exhibit a log2FoldChange > 3 and a basemean normalized expression > 1500.

Ago-bound miRNAs are viewed as the only miRNAs involved in a regulatory process. In contrast free miRNAs not incorporated into an RISC complex are considered non-functional.^2^^,^^3^^,^^4^ As illustrated in Figure 4, we confirmed the existence of both populations of miRNAs (bound or not bound to AGO) in the progenitors of OB interneurons. For this purpose, we used the same T6B x Nestin-CreERT2 transgenic mouse line as in the rest of this manuscript. However, for practical reasons, we performed this comparative experiment four days after tamoxifen injection on T6B-expressing progenitor cells in the subventricular zone (SVZ) rather than on fully mature OB interneurons 34 days after T6B induction. The reason for that is that progenitor cells are much easier to dissociate and FACS sort than mature neurons.

The possibility offered by the Ago-APP technique to specifically analyse the Ago-bound miRNAs makes this approach more accurate in describing the functionality of the miRNAs pathway than sequencing the entire population of expressed miRNAs. Moreover the in vivo Ago-APP technique allows to focus the analysis on a specific cell type, defined by the use of specific Cre drivers, and avoids the need to dissociate the cells of interest. Altogether these advantages make Ago-APP a technique of choice to understand the role of miRNAs in regulating a specific process, particularly during development.

In the associated primary research manuscript,^1^ we also described the generation of another transgenic mouse line in which the T6B-YFH transgene is fused to the PSD95 tag protein. Using this line, we applied the same in vivo Ago-APP technique to decipher the regulatory activity of miRNAs at post-synapse, where they are known to play a very important and specific role in the regulation of plasticity at the level of the individual synapse. This unique possibility opens the way to study the molecular control of synaptic plasticity by miRNAs in specific neurons in response to environmental changes and/or during the achievement of a specific behavioral task by a living animal.

## Limitations

In contrast to immunoprecipitation-based method using anti-argonaute antibodies, the use of T6B to catch active miRNAs does not allow the co-isolation of target mRNAs. Moreover, while the presented sequencing method allows to sequence low amount of material it is not compatible with single-cell miRNA sequencing.

The in vivo Ago-APP technique presented here requires transgenic animals. In addition to the T6B transgene, these animals also harbour a Cre-expressing transgene to induce T6B expression in specific cells. However, for certain cell types, the relevant Cre transgenic mouse line may not be available, in which case it needs to be created, which is a significant undertaking. Therefore, it would be interesting to evaluate the possibility of overcoming this limitation by performing Ago-APP in vivo on tissue in which T6B is transiently expressed, for example through the viral infection of wild-type animals. This would make the protocol broadly versatile across model systems.

## Troubleshooting

### Problem 1

Tissue pieces are not lysed properly after sonication (related to step4).

### Potential solution

• Repeat the sonication process for complete homogenization.
• If working with cells or non-fixed samples try to homogenize with a Dounce homogenizer.

### Problem 2

AGO-APP pulls down a very low amount of miRNA (related to step 5-12).

### Potential solution

• Increase the quantity of input lysate over the 500 μg capacity of ChromoTek’s GFP-Trap® Agarose. Increase it up to 2,000 μg.

### Problem 3

After polyadenylation of miRNA in library prep, there is no miRNA pellet (blue in color because of glycoblue) (related to step 14).

### Potential solution

• Enhance the concentration of NaCl up to 10M.
• Increase the incubation time for miRNA precipitation from 1 h to 3 h at −80°C or Overnight at −20°C.

### Problem 4

Upon PCR amplification of sequencing library only a 142 bp band is amplified (related to step 15-18).

### Potential solution

• Lower the 5′ adapter concentration (Figure 3).

## Resource availability

### Lead contact

Further information and requests for resources and reagents should be directed to and will be fulfilled by the lead contact, Christophe Beclin (christophe.beclin@univ-amu.fr).

### Technical contact

Technical questions on executing this protocol should be directed to and will be answered by the technical contact, Surbhi Kapoor (surbhilal96@gmail.com).

### Materials availability

This manuscript does not generate any new material.

### Data and code availability

This manuscript does not generate any new dataset or code.

## Acknowledgments

This work has been supported by the 10.13039/501100001665ANR (MicroRNAct, ANR-17-CE16-0025; Miniature, ANR-21-CE13-0003) and the 10.13039/501100002915Fondation pour la Recherche Médicale (Programme équipe 10.13039/501100002915FRM). S.K. received a PhD fellowship from Neuroschool Marseille from 10.13039/100007586Aix-Marseille University. A.E. received a post-doc fellowship from the Swiss National Science Foundation (SNSF).

We thank Lena Vilvandre for animal genotyping and the animal facilities of IBDM.

## Author contributions

C.B.: conceptualization, bioinformatic analyses, and writing; S.K.: performing the experiments and writing; A.E.: performing the experiments; H.C.: conceptualization and writing.

## Declaration of interests

The authors declare no competing interests.
